# Water-Use Strategies and Plasticity of Sand-Fixing Plants Along the Tarim Desert Highway in the Taklamakan Desert

**DOI:** 10.3390/plants15213308

**Published:** 2026-10-26

**Authors:** Xiantao Wang, Zhaojie Chen, Xiangru Liu, Xue Wu, Bota Bahethan, Cuiping Tian, Xianyao Yang, Qianqian Yang

**Affiliations:** 1 College of Ecology and Environment, Xinjiang University, Urumqi 830017, China; wangxiantao2025@163.com (X.W.); 107556522298@stu.xju.edu.cn (Z.C.); bota119@xju.edu.cn (B.B.); tcp0827@163.com (C.T.); 107552201192@stu.xju.edu.cn (X.Y.); yangqq@stu.xju.edu.cn (Q.Y.); 2 Key Laboratory of Oasis Ecology, Ministry of Education, Xinjiang University, Urumqi 830017, China; 3 Geological Bureau, Xinjiang Uyghur Autonomous Region, Urumqi 830000, China; 4 Observation and Research Station of Soil and Water Processes and Ecological Security of Oasis in the Headstream Area of the Tarim River, Ministry of Natural Resources, Urumqi 830063, China; lmq0123456@outlook.com

**Keywords:** plant water source, hydrogen and oxygen stable isotope, Bayesian mixture model (MixSIAR), water ecological niche, arid desert ecosystem

## Abstract

In the hyper-arid Taklamakan Desert, where water availability is extremely limited, key sand-fixing species such as *Tamarix ramosissima*, *Calligonum arborescens*, and *Haloxylon ammodendron* are largely maintained by saline groundwater irrigation. Understanding their water-use strategies is therefore essential for optimizing irrigation practices and species selection in shelterbelt systems. However, the water sources, long-term water-use efficiency (WUE), and interspecific water competition of these species across different establishment years remain insufficiently understood. The primary objective of this study is therefore to elucidate the water-use strategies of these three sand-fixing species and to assess how they vary with stand age and mediate interspecific coexistence. To achieve this objective, stable hydrogen, oxygen, and carbon isotope analysis (*δ*^2^H, *δ*^18^O, and, *δ*^13^C) was integrated with MixSIAR modeling and water niche assessment for stands established in 1999, 2004, and 2009. The results showed that: (1) water sources differed significantly among species and changed dynamically with stand age, with *C. arborescens* exhibiting the most pronounced variation in water-uptake depth and the greatest water-use plasticity; (2) long-term WUE varied significantly across species, and its relationship with water-niche breadth showed a negative trend, suggesting that broader water-niche breadth may be associated with lower WUE; and (3) interspecific water competition diminished as increasing plantation age increased. These findings demonstrate that in the extreme arid environment of the Taklamakan Desert, sand-fixing species achieve coexistence through divergent water-use strategies, thereby mitigating competition under severe water scarcity. This study provides a theoretical foundation for the sustainable management of shelterbelt ecosystems in arid regions.

## 1. Introduction

Within the soil–plant–atmosphere continuum (SPAC), precipitation, groundwater, and soil water constitute the principal potential water sources for plants [1,2]. Among these, soil water serves as the most direct and central source for plant uptake, playing an irreplaceable role in plant physiology and ecosystem water cycling [3]. Stable hydrogen and oxygen isotope techniques provide a powerful tool for tracing plant water sources. The approach is based on the principle that isotope fractionation is negligible during root water absorption and transport in plants, meaning the *δ*^2^H and *δ*^18^O values of xylem water directly reflect the isotopic signatures of the soil water being utilized [4]. When distinct potential water sources exhibit sufficiently different isotopic signatures, this method can effectively reveal plant water-use strategies [5,6,7]. Due to its low invasiveness and high precision, the technique has been widely employed in studies of plant–water relations in arid and semi-arid regions [8,9,10].

Stable hydrogen and oxygen isotope techniques have revealed a spectrum of water-use strategies among plants in arid regions. For instance, Wang et al. found that the water sources for *Tamarix ramosissima* shift in response to precipitation patterns [9]. In desert steppe ecosystems, large shrubs tend to rely on stable deep soil water or groundwater, while shallow-rooted semi-shrubs and herbs often exhibit more flexible water-use patterns to cope with environmental fluctuations [11]. More studies showed significant interspecific differences in the proportional use of precipitation, groundwater, and soil water, reflecting divergent water-use strategies among coexisting species [12,13]. These differences can be further interpreted through the framework of ecological niche theory. Quantitative assessments of niche breadth and overlap provide a valuable perspective for understanding water partitioning within plant communities [14]. For instance, Liu et al. reported that greater shrub encroachment leads to increased water-niche overlap, which in turn reduces community stability [15,16]. In arid and semi-arid ecosystems, plants often adapt to spatiotemporal heterogeneity through water-niche segregation [17]. Consequently, coexisting species exhibit distinct water-use patterns, which helps mitigate competition for water and promotes community stability [17,18]. Stable isotope-based studies confirm that water niche segregation is a common phenomenon among plants in arid lands [19]. This differential utilization of water sources significantly reduces interspecific competition and serves as a key mechanism for maintaining species coexistence and community stability.

The Taklamakan Desert is an extremely arid region with an average annual precipitation of less than 50 mm, where the ecological environment is highly fragile [20]. To promote regional transportation and resource development, the Taklamakan Desert Highway (Luntai–Minfeng section) was constructed in 1993 [21]. To ensure the road remains accessible under severe wind-sand conditions, a large-scale shelterbelt project was established along both sides of the highway, which still plays a strategically irreplaceable role in regional socio-economic development [22]. The shelterbelt extends 436 km in length, with a width of 72–78 m and a total area of 3128 ha. It is primarily planted with highly stress-tolerant sand-fixing shrubs, including *Tamarix ramosissima* Ledeb, *Calligonum arborescens* Litv and *Haloxylon ammodendron* (C.A.Mey.) Bunge. They are maintained by drip irrigation using highly mineralized groundwater. Previous research has shown that the establishment of this shelterbelt has effectively stabilized mobile dunes and significantly improved the ecological conditions along the highway [23].

However, most existing studies have focused on the ecological benefits of the shelterbelt. For this type of artificial ecosystem that depends on saline-water irrigation, it remains unclear how the water-use strategies and competitive relationships among different plant species evolve over time. Against this background, key scientific and management questions emerge: Under long-term and consistent irrigation management, do the water-use strategies of these sand-fixing plants diverge? How does their water competition change with planting age? To address these questions, we selected three sand-fixing species (*T. ramosissima*, *C. arborescens* and *H. ammodendron*) established in different years (1999, 2004, and 2009). We measured stable carbon isotopes in plant assimilation branches (leaf degeneration in order to adapt to arid environments), measured stable hydrogen and oxygen isotopes in xylem water and sub-canopy soil water, performed plant water-source analysis, and calculated long-term water-use efficiency (WUE) and water-niche characteristics. The aim of this study is to comprehensively assess the water-use strategies of these three sand-fixing species by integrating water sources, long-term WUE, water-niche breadth, interspecific water competition, and water-use plasticity across different planting years, and thereby to evaluate water-niche complementarity and competition among them. These findings will provide a theoretical basis for selecting optimal species combinations and enhancing the WUE and long-term stability of shelterbelt communities in arid regions.

## 2. Results

### 2.1. Characteristics of Soil Water Content Variation

**Figure 1:**
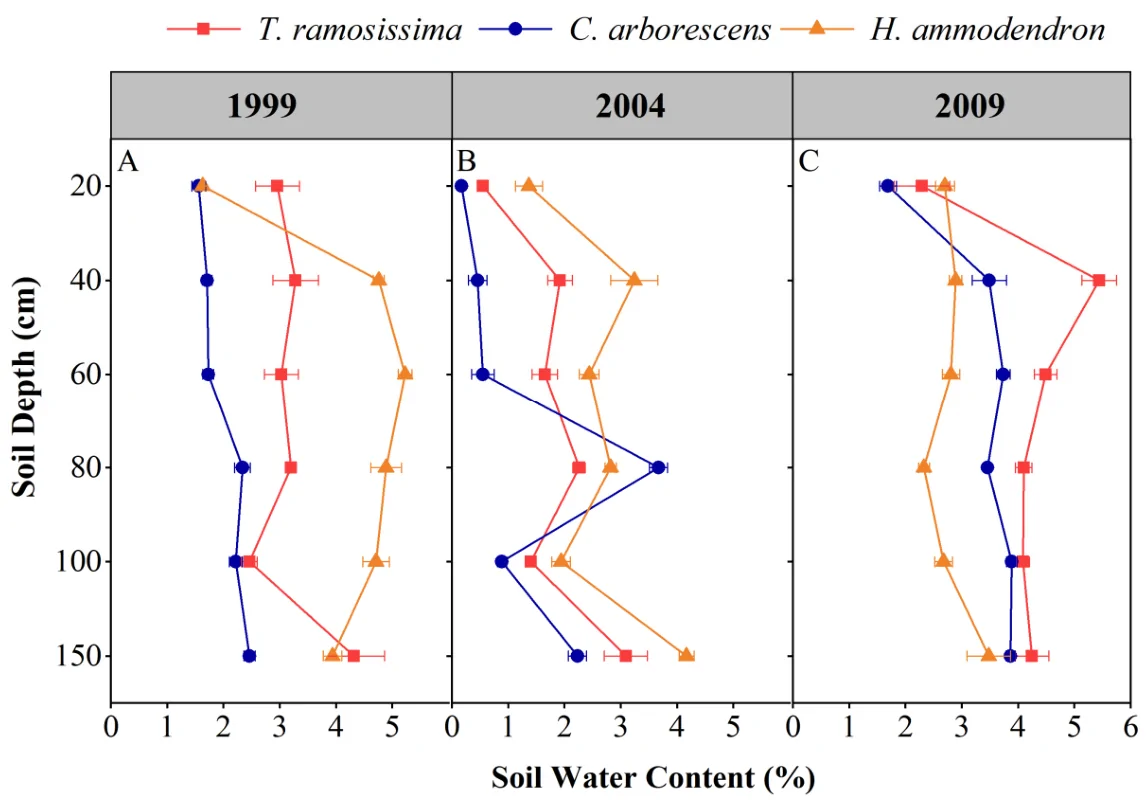
Distribution of soil water content along soil profiles under three sand-fixing plant species (*T. ramosissima*—red squares; *C. arborescens*—blue circles; *H. ammodendron*—orange triangles) across different planting years. (**A**)—1999, (**B**)—2004, and (**C**)—2009.

Across all planting years, the soil water content beneath the canopy of different species was consistently lowest in the surface layer (0–20 cm), generally increased with depth albeit with minor fluctuations, and ranged from 0.143% to 5.95% (Figure 1). Significant differences in soil water content were detected among the three species across planting years (*F* = 17.28, *p* < 0.001). Specifically, the mean soil water content under *T. ramosissima* and *C. arborescens* was highest in 2009, whereas that under *H. ammodendron* reached its maximum in 1999. Within the same planting year, soil water content also differed significantly among species (*F* = 7.74, *p* < 0.01). In 1999 and 2004, *H. ammodendron* exhibited a significantly higher mean under-canopy soil water content than *C. arborescens* and *T. ramosissima*, with the latter showing the lowest values (*p* < 0.05). In contrast, in 2009, the mean soil water content under *T. ramosissima* was significantly greater than that under both *C. arborescens* and *H. ammodendron*, while *H. ammodendron* had the lowest value (*p* < 0.001).

### 2.2. Characteristics of Soil Water δ^18^O

**Figure 2:**
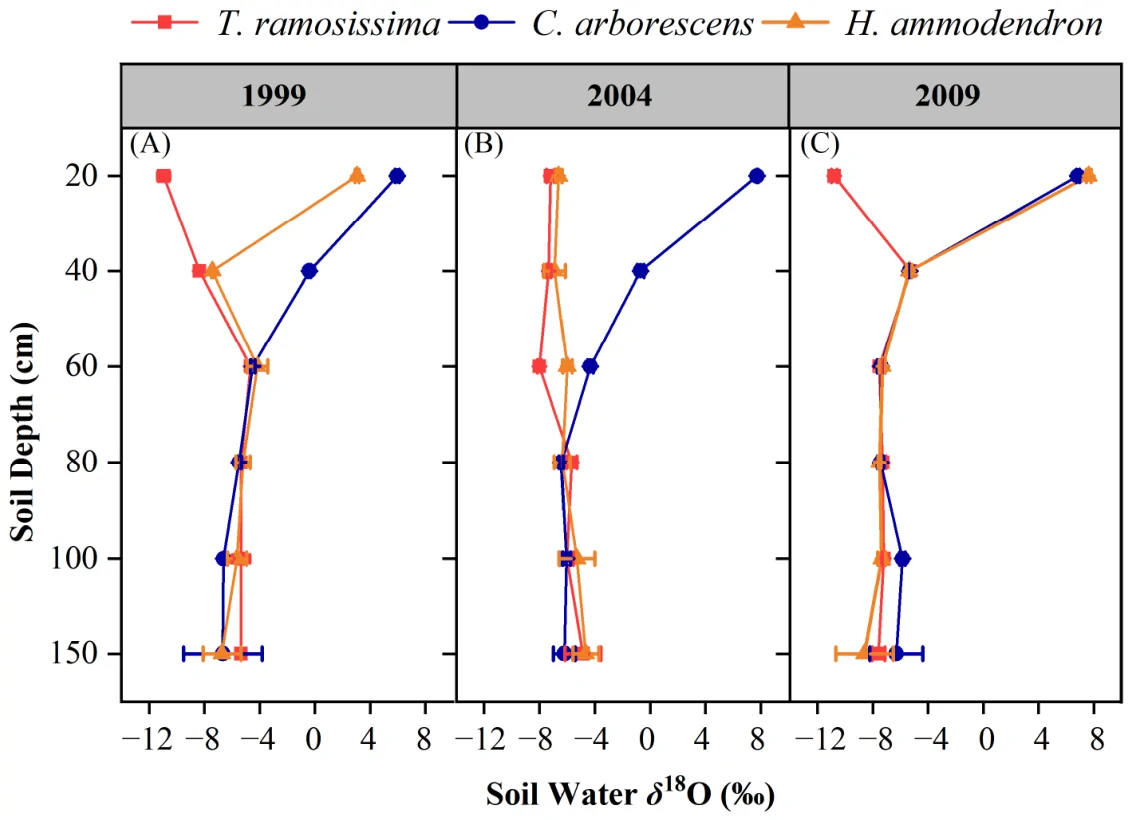
Distribution of soil water *δ*^18^O along soil profiles under three sand-fixing plant species (*T. ramosissima*—red squares; *C. arborescens*—blue circles; *H. ammodendron*—orange triangles) across different planting years. (**A**)—1999, (**B**)—2004, and (**C**)—2009.

Soil water *δ*^18^O exhibited a gradual depletion (i.e., increasingly negative values) with increasing soil depth, ranging from 7.75‰ to −10.94‰. Surface soil water *δ*^18^O was generally enriched in heavy isotopes, with the exception of *T. ramosissima*, under which surface *δ*^18^O remained negative across all three planting years (Figure 2). Two-way ANOVA analysis indicated no significant differences in soil water *δ*^18^O among the three species across planting years (*F* = 0.22, *p* ≥ 0.05); however, significant differences were detected among species within the same planting year (*F* = 4.26, *p* < 0.05).

In 1999, *δ*^18^O in the 0–60 cm soil layer varied markedly among species. The most depleted value (−10.94‰) was observed under *T. ramosissima*, whereas the most enriched values were recorded under *C. arborescens* (5.97‰) and *H. ammodendron* (3.05‰). In 2004, *δ*^18^O in the 0–80 cm layer differed notably between *C. arborescens* and the other two species. *T. ramosissima* and *H. ammodendron* exhibited similar trends, both reaching their minimum values, while *C. arborescens* showed the highest *δ*^18^O (7.75‰). In 2009, pronounced variations in *δ*^18^O occurred in the 0–40 cm layer between *T. ramosissima* and the other two species. The lowest value (−10.79‰) was found under *T. ramosissima*, whereas *C. arborescens* and *H. ammodendron* displayed similar patterns, both approaching their maximum values.

### 2.3. δ^18^O Characteristics of Plant Xylem Water

**Figure 3:**
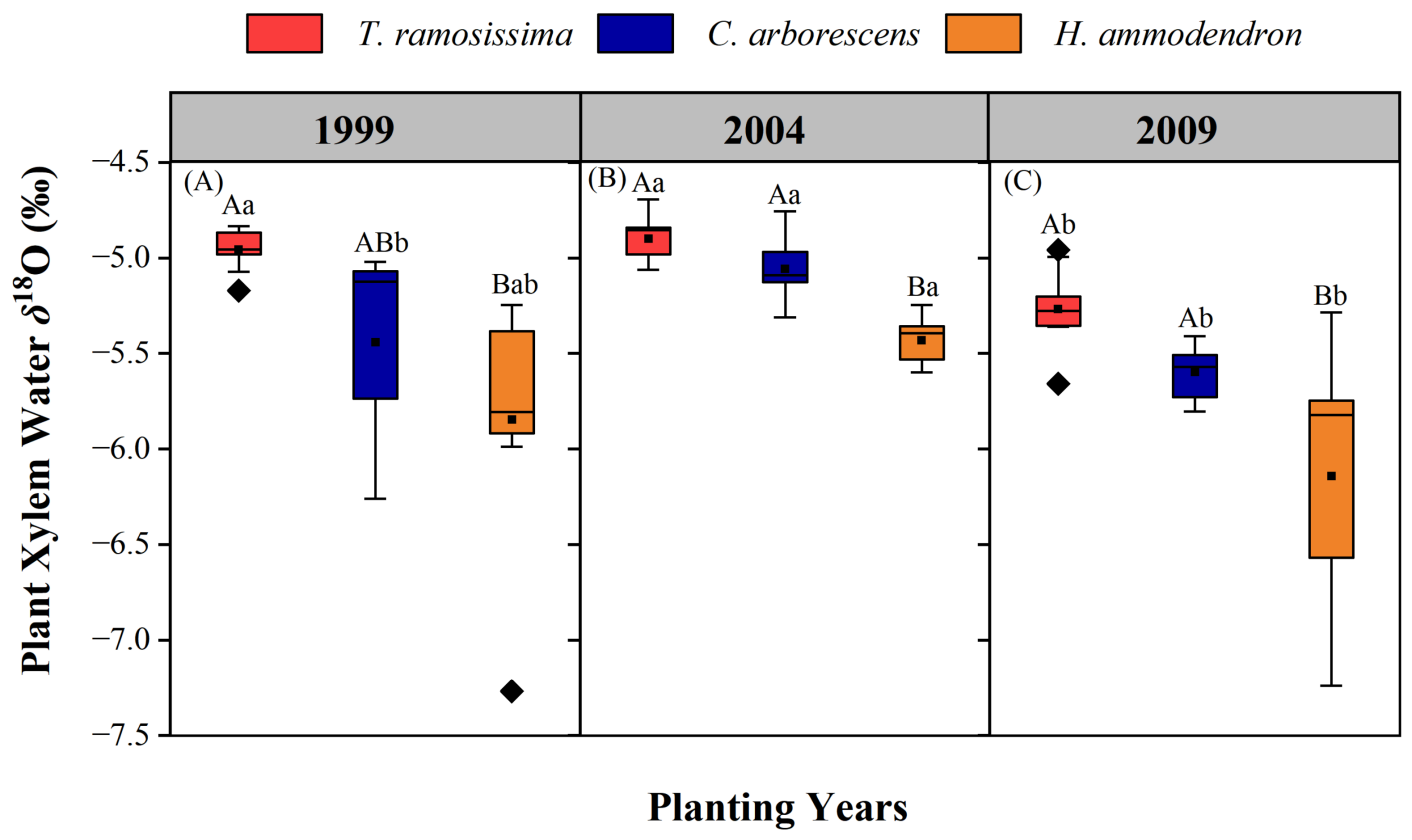
Xylem water *δ*^18^O of three sand-fixing plant species (*T. ramosissima*—red; *C. arborescens*—blue; *H. ammodendron*—orange) across different planting years. (**A**)—1999, (**B**)—2004, and (**C**)—2009. Different uppercase letters for differences among species within the same planting year, different lowercase letters for differences among planting years within the same species.

Xylem water *δ*^18^O across all plants ranged from −7.27‰ to 4.69‰, and showed a progressive depletion (i.e., increasingly negative values) with increasing planting year. The overall variability in xylem water *δ*^18^O was greater for *C. arborescens* and *H. ammodendron* than for *T. ramosissima*. Two-way ANOVA revealed that both planting year and species exerted significant effects on xylem water *δ*^18^O (year: *F* = 15.37, *p* < 0.01; species: *F* = 31.07, *p* < 0.01) (Figure 3). Within the same planting year, *T. ramosissima* exhibited significantly higher *δ*^18^O than *H. ammodendron*, but showed no significant difference from *C. arborescens*. In 2004 and 2009, xylem water *δ*^18^O differed significantly between *C. arborescens* and *H. ammodendron*, with *C. arborescens* displaying higher values. For the same species across planting years, *T. ramosissima* showed no significant difference between 1999 and 2004, but both years differed significantly from 2009. *C. arborescens* exhibited a significant difference in 2004 compared with the other two years, whereas *H. ammodendron* showed a significant difference only between 2004 and 2009.

### 2.4. Water Use Sources of Sand-Fixing Plants in Different Planting Years

**Figure 4:**
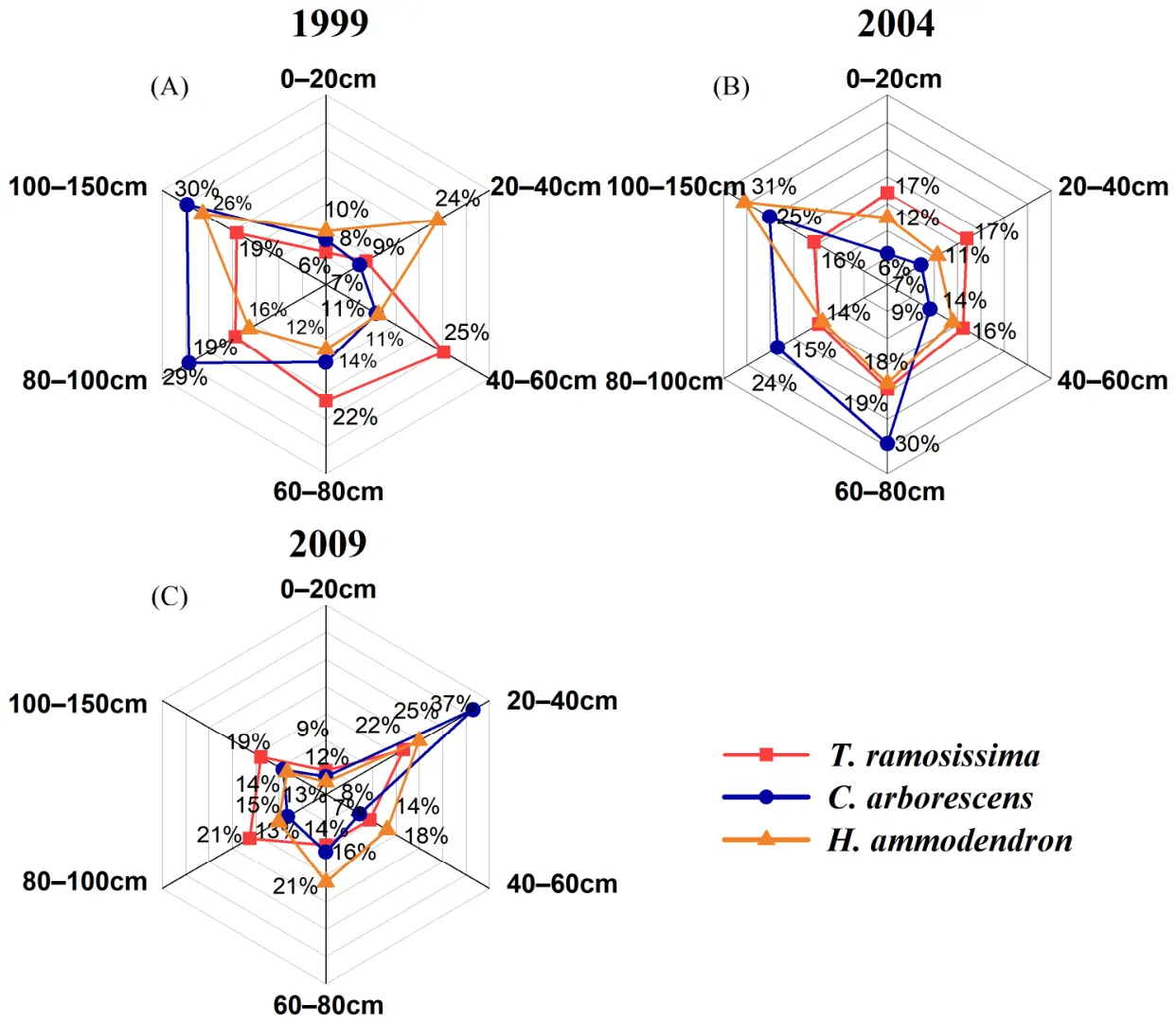
Utilization proportions of potential soil water sources by three sand-fixing plant species (*T. ramosissima*—red line with squares; *C. arborescens*—blue line with circles; *H. ammodendron*—orange line with triangles) across different planting years. (**A**)—1999, (**B**)—2004, and (**C**)—2009.

Based on the MixSIAR model analysis (Figure 4), the water uptake patterns of the three species varied substantially across planting years. In 1999, *T. ramosissima* predominantly extracted water from the 40–60 cm and 60–80 cm soil layers, which together contributed 47% of its total water uptake. *C. arborescens* relied primarily on the 80–100 cm and 100–150 cm layers, accounting for 59% of its uptake, whereas *H. ammodendron* mainly utilized the 20–40 cm and 100–150 cm layers, collectively representing 50%. In 2004, *T. ramosissima* showed a primary dependence on the 60–80 cm layer, with a contribution of 19%. *C. arborescens* predominantly absorbed water from the 60–150 cm depth range, with the highest contribution (30%) occurring in the 60–80 cm layer. *H. ammodendron* exhibited a strong reliance on the 100–150 cm layer, which supplied 31% of its water uptake. In 2009, *T. ramosissima* primarily utilized the 20–40 cm layer (22%), while *C. arborescens* preferentially extracted shallow soil water from the same depth interval (37%). In contrast, *H. ammodendron* displayed a relatively uniform water uptake pattern across the 20–80 cm depth range. Over the chronosequence, the primary water uptake depth of all three species gradually deepened, with *C. arborescens* exhibiting the most pronounced shift in its dominant water-absorbing layers over time.

### 2.5. WUE and Water Niche Breadth Index of Sand-Fixing Plants in Different Planting Years and Their Correlation

The mean *δ*^13^C values for *T. ramosissima*, *H. ammodendron*, and *C. arborescens* across the planting years were −26.70‰ ± 0.27‰, −15.62‰ ± 0.36‰, and −15.18‰ ± 0.18‰, respectively. Correspondingly, their mean long-term water use efficiency (WUE) was 96.33 ± 2.56, 221.98 ± 2.77, and 229.73 ± 2.08 μmol CO_2_ mol^−1^ H_2_O, respectively.

**Figure 5:**
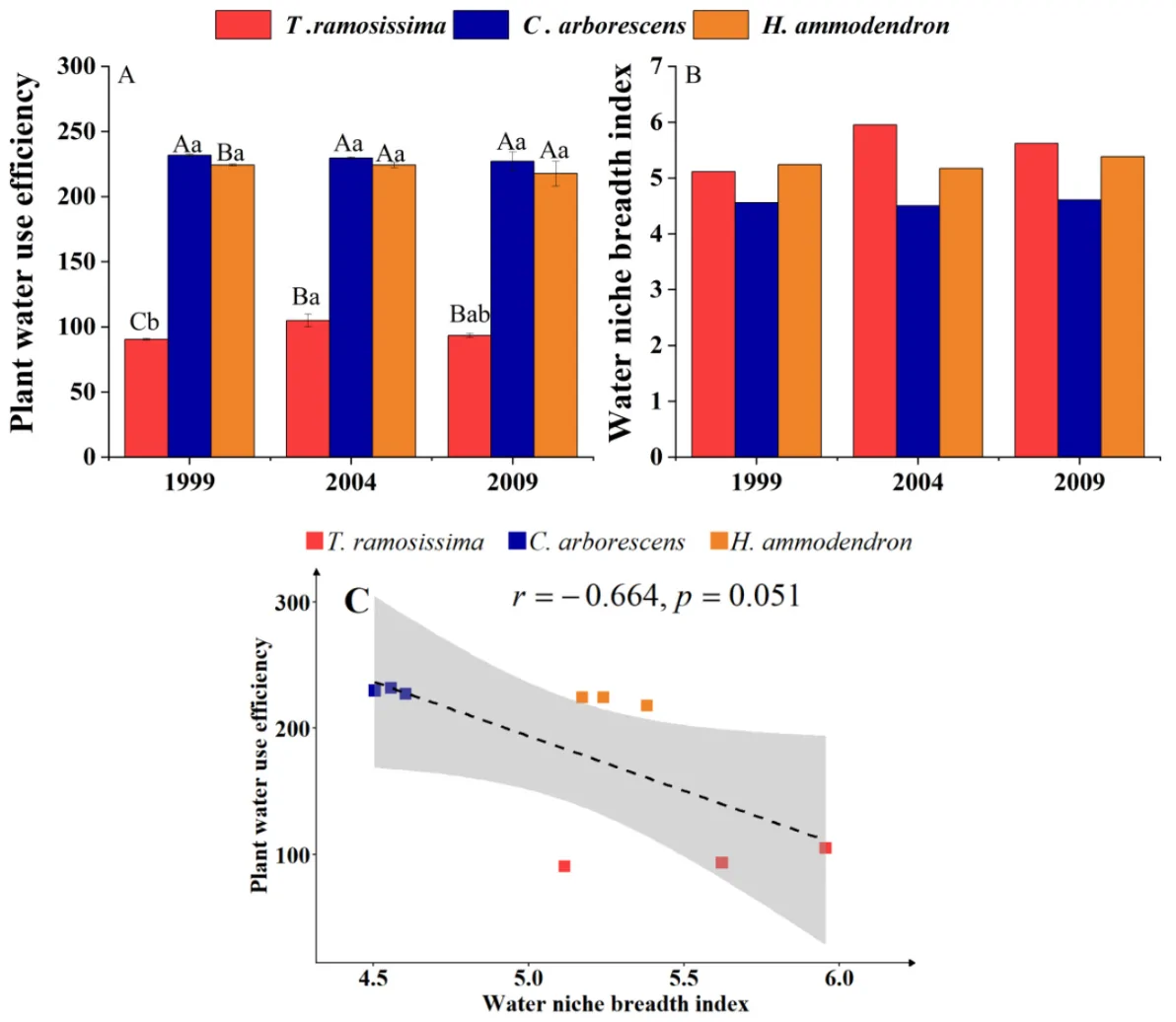
Plant water use efficiency (WUE), water niche breadth index, and their relationship among three sand-fixing plant species (*T. ramosissima*—red; *C. arborescens*—blue; *H. ammodendron*—orange) across different planting years. (**A**)—plant WUE; (**B**)—water niche breadth index; (**C**)—relationship between plant WUE and water niche breadth index. Different uppercase letters indicate differences among species within the same planting year; different lowercase letters indicate differences among planting years within the same species. The gray area represents the 95% confidence interval.

Two-way ANOVA (Figure 5A) revealed that species identity exerted a highly significant effect on WUE (*F* = 292.66, *p* < 0.001). Within each planting year, *T. ramosissima* exhibited significantly lower WUE than both *H. ammodendron* and *C. arborescens*. In 1999, WUE differed significantly among all three species. However, in 2004 and 2009, no significant difference in WUE was detected between *H. ammodendron* and *C. arborescens*. For the same species across years, *T. ramosissima* showed a significant difference in WUE between 1999 and 2004.

Water niche breadth indices, calculated from water source contributions derived from the MixSIAR model, ranged from 4.505 to 5.955 across all species (Figure 5B). In 2004, *T. ramosissima* showed the widest water niche, whereas *C. arborescens* had the narrowest. The water niche breadth of *C. arborescens* and *H. ammodendron* remained relatively stable across years. Overall, long-term WUE declined with the increasing water niche breadth. A moderate negative correlation was observed (r = −0.664), but it did not reach the conventional threshold for statistical significance (*p* = 0.051), since *p* > 0.05 (Figure 5C).

### 2.6. Proportion Similarity Index of Water Use Among Different Sand-Fixing Plant Species

**Figure 6:**
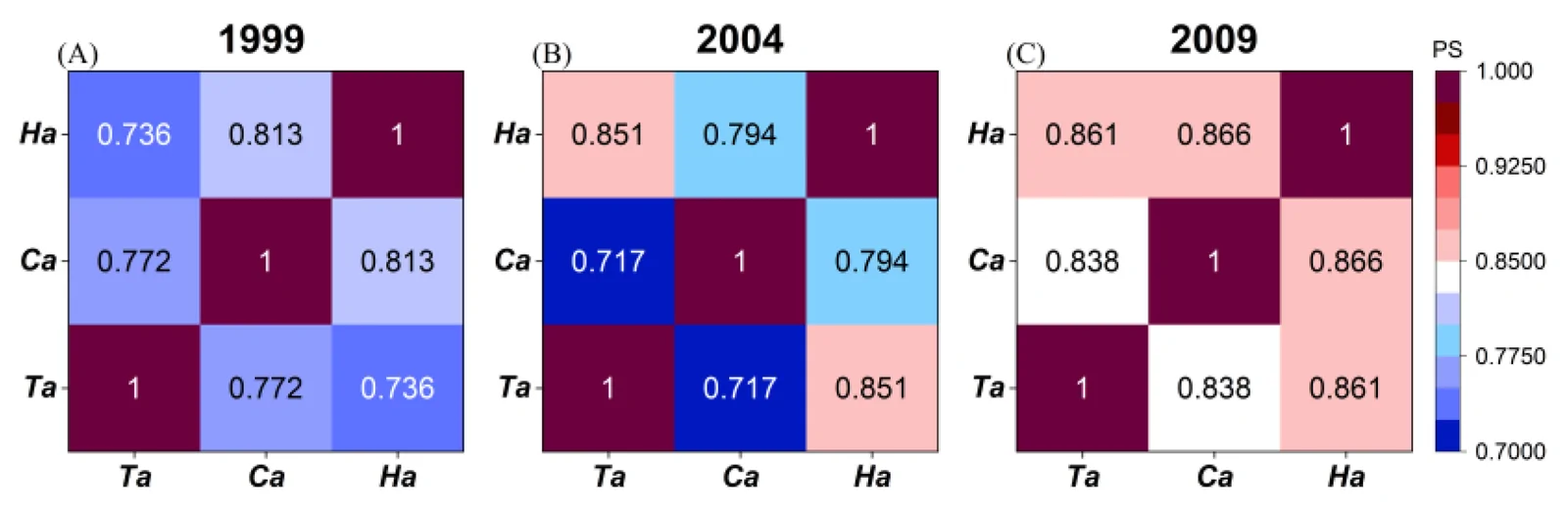
Proportional Similarity (PS) index of water use among three sand-fixing plant species (*T. ramosissima*—Ta; *C. arborescens*—Ca; *H. ammodendron*—Ha) across different planting years. (**A**)—1999, (**B**)—2004, and (**C**)—2009.

The PS was employed to quantify the degree of water-niche overlap among the three sand-fixing species (Figure 6). PS values ranged from 71.7% to 86.6% across all planting years. The highest mean PS was observed in 2009, indicating the most intense water competition among plants established in that year. In contrast, the lowest PS variability was found among species planted in 1999, suggesting a relatively lower level of interspecific competition.

## 3. Discussion

### 3.1. Soil Water Oxygen Isotope Composition Characteristics Under the Canopies of Three Sand-Fixing Plants

In this study, the *δ*^18^O values of surface soil water at 0–20 cm and 20–40 cm were higher for all three sand-fixing plants, indicating strong evaporation and significant isotopic fractionation in the surface soil [24,25,26]. As soil depth increased to 80–100 cm and 100–150 cm, the variation in *δ*^18^O decreased and stabilized. This is consistent with previous findings by Zhu et al. that the influence of evaporation on stable isotopes in soil water diminishes with increasing depth [27]. Consistently, Chen et al. also reported that the enrichment of *δ*^18^O in surface soil water results from direct exposure to evaporative conditions [28]. As soil depth increases, evaporation effects diminish, leading to gradual *δ*^18^O depletion, with smaller fluctuations observed in deeper soil layers. This vertical differentiation reflects the depth-dependent response of soil water isotopic signatures to environmental processes.

From a species-specific perspective, the intrinsic root distribution traits of the three sand-fixing plants are the primary factors influencing soil water *δ*^18^O composition under their canopies. Notably, these inherent traits exert a long-term regulatory effect that outweighs the impacts of short-term seasonal growth cycles. Deep-rooted plants like *T. ramosissima* transport depleted isotopes from deep layers to the surface through hydraulic lifting [29,30], diluting evaporation-induced enrichment effects and resulting in more negative *δ*^18^O values in surface soil water across all three planting years. In contrast, the surface soil water *δ*^18^O of *C. arborescens* and *H. ammodendron* generally showed a positive trend, with both species exhibiting consistent variation patterns. Although ANOVA indicated no significant differences in soil water *δ*^18^O across different planting years, *H. ammodendron* still exhibited certain fluctuations in *δ*^18^O values.

### 3.2. Xylem Water δ^18^O Signatures and Interspecific Differences in Water Sources Among Three Sand-Fixing Plant Species

Significant differences were observed in *δ*^18^O of xylem water among *T. ramosissima*, *C. arborescens*, and *H. ammodendron*, with a general depletion trend over planting years. These variations likely correlate closely with the root distribution characteristics of different plants and the isotopic composition of soil water absorbed by each species. In line with the vertical profile of soil water *δ*^18^O, the main water uptake layers for all three species also showed progressive isotopic depletion over time, consistent with the shift in xylem water *δ*^18^O. For instance, at the 100–150 cm soil layer, soil water *δ*^18^O for *T. ramosissima* decreased from −5.35‰ in 1999 to −7.58‰ in 2009, and under *H. ammodendron* from −6.72‰ to −8.58‰, with a similar trend occurring under *C. arborescens*. Further analysis of plant water uptake strategies across different planting years revealed that *T. ramosissima* and *C. arborescens* planted in 2004 exhibited higher proportions of soil water utilization from the 80–100 cm soil layer (19.37% and 29.50%, respectively), and their xylem water *δ*^18^O (−5.68‰) closely matched soil water *δ*^18^O (−6.43‰) in that layer. In contrast, *H. ammodendron* predominantly absorbed water from the 150 cm soil layer, with a contribution ratio of 30.633%, corresponding to a soil water *δ*^18^O value of −4.651‰, indicating a downward shift in its water uptake zone and an enrichment in heavier isotopes. By 2009, the proportion of soil water utilized by *H. ammodendron* at 60–80 cm reached 21.27%, and that by *T. ramosissima* at 20–40 cm reached 21.61%. Soil water isotopes in this range were relatively depleted, consistent with the further decrease in *δ*^18^O of *H. ammodendron* xylem water.

The above changes align with patterns revealed by multiple studies: with increasing stand age, plants often transition from initial reliance on shallow soil water to greater utilization of deep soil water [31,32,33,34]. For instance, in the Heihe River Basin, mature perennial *Tamarix* spp. significantly increased its utilization of deep groundwater; at the southern edge of the Taklamakan Desert, *H. ammodendron* gradually shifted its water uptake to soil layers below 100 cm with increasing stand age; and in the Mu Us Desert, *Caragana korshinskii* deepened its soil water utilization depth with increasing stand age [35]. Collectively, these cases demonstrate that plants develop dynamically adaptive water-use strategies throughout their ontogeny: while possessing the capacity to access deep soil water, their actual uptake depth flexibly adjusts in response to soil moisture conditions [36]. This mechanism is consistent with the framework proposed by Wang et al. [37], in which interspecific differences in uptake depth generate isotopic divergence in xylem water, further demonstrating that plants in dryland environments dynamically modulate their uptake depth and source proportions in concert with changes in soil moisture.

Although *T. ramosissima*, *C. arborescens* and *H. ammodendron* occupy the same habitat and face similar potential water sources, their primary water-absorbing soil layers exhibit distinct differences [38]. *T. ramosissima* displays typical root dichotomy, featuring a “herringbone-like” root structure characterized by both a well-developed vertical taproot and an extensively spreading lateral root system [39]. This root configuration confers flexible water-use adaptation, enabling facultative utilization of both shallow and deeper water sources beyond the extremely arid topsoil layer (0–20 cm). *C. arborescens* exhibits highly developed horizontal roots but relatively limited vertical rooting capacity, classifying it as a typical shallow-rooted plant. During the early establishment phase (2009), its water uptake was significantly concentrated in the shallow soil layer (20–40 cm, 36.5%), fully reflecting its shallow-rooted nature. By the mid-establishment phase (2004), the primary water-absorption zones shifted downward to 60–80 cm (29.5%) and 80–100 cm (23.5%), reflecting expanded utilization of mid-depth moisture as growth progressed; by the late establishment stage (1999), the plant primarily utilized deep soil moisture at 80–100 cm (29.3%) and 100–150 cm (29.7%). As a typical deep-rooted plant, *H. ammodendron* possesses a long taproot and extensive lateral roots, enabling efficient utilization of deep soil water and even groundwater. Across all plantation age classes, the 100–150 cm depth interval remained the dominant water source, reflecting a sustained dependence on deep-seated soil moisture conferred by the *H. ammodendron* deep-rooted architecture.

### 3.3. Water Niche and Water Source Competition Among Three Sand-Fixing Plants

Both WUE and water niche breadth exhibited significant species specificity. Water niche breadth reflects a species’ environmental adaptability and water resource utilization efficiency [40]. The Levins index shows that *T. ramosissima* has the largest average water niche breadth, corresponding to its flexible water uptake across different soil depths, allowing it to utilize water from various sources in response to fluctuating moisture availability. This aligns with the observation of Li et al. [41] that desert plants with broad niches possess greater plasticity in multi-source water utilization. In contrast, *C. arborescens* exhibited a narrower niche width that varied little across planting years, indicating a conservative and specialized water-use strategy. In terms of WUE, *C. arborescens* and *H. ammodendron* showed higher WUE, consistent with their less negative *δ*^13^C, reflecting the characteristic high water efficiency of the C_4_ photosynthetic pathway. In contrast, *T. ramosissima*, as a C_3_ plant, exhibited lower WUE, a higher water-consumption intensity, and a greater dependence on water availability. A negative correlation trend between niche breadth and long-term WUE was observed in this study (*p* = 0.051), although it did not reach the conventional threshold for statistical significance (*p* < 0.05), possibly owing to insufficient statistical power due to the limited sample size. This suggests that wider niche breadth may be associated with lower WUE.

The PS reflects resource utilization similarity among species, with higher values indicating greater overlap in water sources, which may imply stronger potential competition [42,43]. When examined along the chronosequence from recent to older plantations, the PS indices of the three species pairs displayed a distinctive pattern: they initially remained high, subsequently fluctuated with progressive differentiation, and eventually declined overall. In 1999, the PS indices of all three species pairs remained within a low range, with *T. ramosissima*–*H. ammodendron* showing the lowest value and *C. arborescens*–*H. ammodendron* exhibiting the highest, indicating a relatively low degree of water-source overlap. A possible explanation is that, in the later stages of establishment, plant root systems had fully developed and may have partitioned soil moisture at different depths, thereby reducing overlap in water uptake. This pattern is consistent with the findings of Li et al. that desert plants exhibit significant root stratification in the later stages of establishment, leading to reduced water competition intensity [40].

In 2004, the PS index exhibited pronounced interspecific differentiation. The *T. ramosissima*–*C. arborescens* pair recorded the lowest values across all three planting years. In contrast, the *C. arborescens*–*H. ammodendron* pair reached 0.794, a slight decline from 1999, whereas the *T. ramosissima*–*H. ammodendron* pair increased sharply to 0.851, representing the largest interspecific change among the three species pairs during this period. In 2009 (early establishment), PS indices for all species pairs had markedly increased compared to 1999, reaching peak values across all three planting years. The *C. arborescens*–*H. ammodendron* pair exhibited the highest index, followed by the *T. ramosissima*–*H. ammodendron* pair, while the *T. ramosissima*–*C. arborescens* pair showed the lowest index, indicating a further increase in water-source overlap among species. During this phase, plant root systems were not yet fully developed and appeared to rely mainly on readily accessible surface water sources. This corresponded to highly convergent water uptake patterns, which may reflect greater potential competition for water. Overall, the temporal dynamics of the PS index from 1999 to 2009 primarily reflect shifts in the degree of water-source overlap, which may be linked to changes in root development and water uptake depth with plantation age. This change aligns with the perspective proposed by Huo et al. that forest age influences plant water use overlap by regulating root distribution depth [33].

In summary, the degree of water-source overlap among species varied over the chronosequence and was accompanied by shifts in water uptake depth. When faced with changes in water availability induced by different planting ages, the three sand-fixing plants exhibit distinct water source use: *T. ramosissima* used a broader range of water sources, while *H. ammodendron* and *C. arborescens* maintained relatively stable water-source use, coupled with high WUE. With increasing plantation age, changes in water demand associated with plant growth may have contributed to shifts in the degree of water-source overlap. Over time, the observed divergence in water uptake depth may reflect a pattern of niche differentiation [44,45]; however, whether this alleviates competition and facilitates coexistence cannot be directly inferred from the present data and requires further investigation.

## 4. Materials and Methods

### 4.1. Study Area

**Figure 7:**
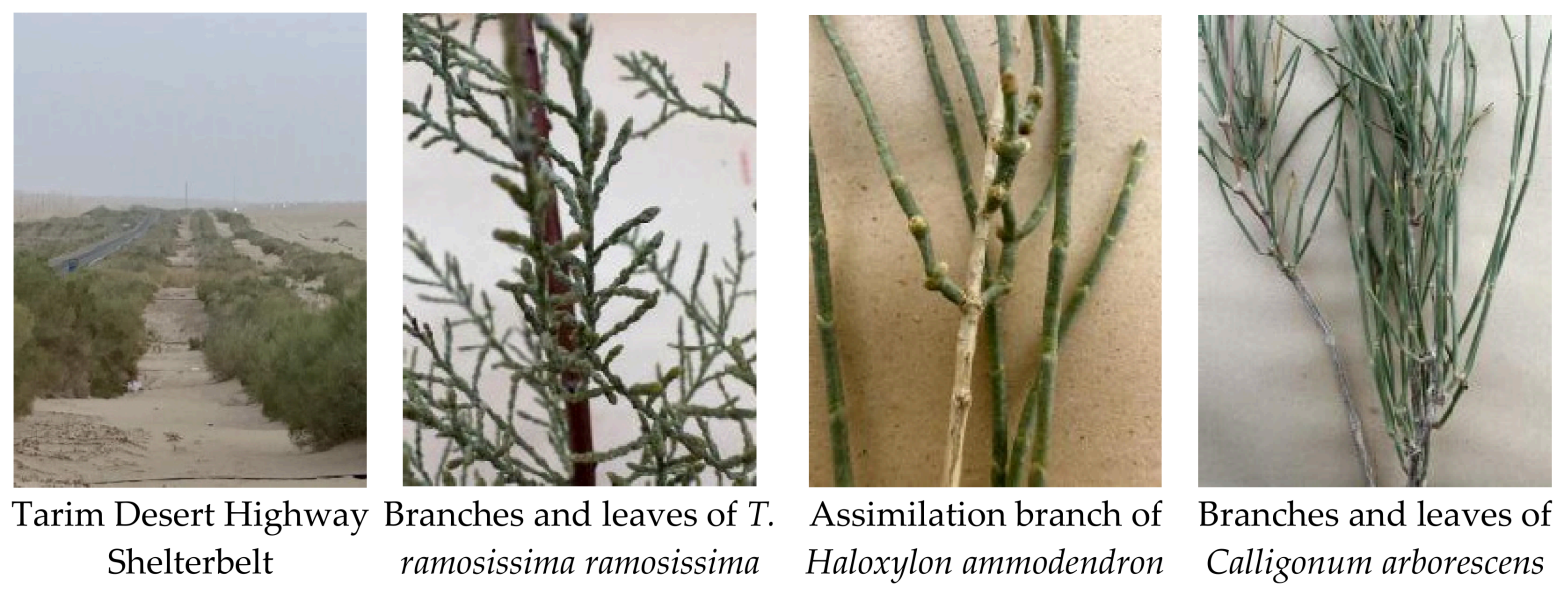
Desert highway shelterbelt and the assimilating branch three sand-fixing species.

The study area is located in the Tarim Desert Highway Shelterbelt (82°45′–84°15′E, 38°15′–40°30′ N) in the hinterland of the Taklimakan Desert in Xinjiang, China. This region is characterized by a typical temperate hyperarid desert climate, with annual precipitation less than 50 mm that has a highly uneven spatiotemporal distribution, concentrated primarily between June and August. The annual evaporation reaches 2800–3200 mm, and the aridity index exceeds 50. The annual average temperature is 12.4 °C, with summer extremes reaching 45 °C and winter lows dropping to −25 °C. Daily temperature fluctuations often exceed 20 °C. Sandstorm activity is frequent, with average annual wind speeds of 2.5–4.5 m/s and over 100 days of dust storms. The predominant soil type is aeolian sandy soil, characterized by loose texture and poor water retention. Soil pH ranges from 7.5 to 9, exhibiting strong alkalinity. Surface vegetation is sparse, with natural cover dominated by drought-tolerant species such as *Alhagi sparsifolia* and *Calligonum* spp. Artificial shelterbelts primarily line roadways, consisting mainly of *T. ramosissima*, *C. arborescens* and *H. ammodendron* (Figure 7). These species exhibit outstanding drought tolerance, cold resistance, salt tolerance, and wind-sand resistance, making them crucial ecological species for windbreak construction, sand fixation, and desertification mitigation. The shelterbelts are maintained by drip irrigation using highly mineralized groundwater (with a range of 2.8–29.78 g/L) along the route. Irrigation is applied 2–3 times per month, with approximately 15 L per plant per irrigation event.

### 4.2. Field Investigation and Sample Collection

#### 4.2.1. Plant Samples Collection and Processing

The predominant planting years of the shelterbelts (1999, 2004, and 2009) were identified through an extensive literature review and corroborated by field interviews with local forest rangers. For each planting year, one representative sample plot was established, within which three 10 × 10 m quadrats were set up. Within each quadrat, standard vegetation surveys were performed to record key structural attributes, including crown width, plant height, and basal diameter.

During the peak growing season, well-developed and morphologically similar sand-fixing plants were selected from each plot. Lignified twig samples were collected from branches oriented in multiple directions. For each individual, a segment of approximately 3–4 cm in length was excised using pruning shears. Following the removal of the phloem, each sample was immediately placed into an 8 mL glass vial, sealed with Parafilm, and temporarily preserved on ice in a portable cooler for transport. In the laboratory, all samples were stored at below –20 °C until stable hydrogen and oxygen isotope analyses (*δ*^2^H and *δ*^18^O) were conducted.

Simultaneously, assimilation shoot samples were collected from the same plants, placed in paper envelopes, labeled, and transported to the laboratory [8]. Upon arrival, samples were oven-dried at 105 °C for 30 min to deactivate enzymes, followed by drying at a constant temperature of 65 °C until reaching a constant mass. The dried materials were then ground to pass through a 100 mesh sieve and subsequently transferred into sample tubes. Throughout the entire preparation process, strict measures were implemented to prevent cross-contamination. The prepared samples were finally used for stable carbon isotope composition (*δ*^13^C) analysis.

#### 4.2.2. Soil Samples Collection and Processing

Prior to soil sampling, surface litter was carefully removed from beneath each plant canopy. Soil cores were then collected using a stainless steel auger (5 cm inner diameter) at six depth intervals: 0–20, 20–40, 40–60, 60–80, 80–100, and 100–150 cm. Visible roots and gravel were manually removed from each core. Each layer’s soil was thoroughly homogenized and subsampled into three aliquots for distinct purposes: (1) oven-drying in aluminum containers for gravimetric soil moisture content determination; (2) storage in resealable plastic bags for analyses of soil physicochemical properties; and (3) storage in resealable bags for stable isotope ratio measurements (*δ*^2^H and *δ*^18^O). Three replicate cores were collected per depth layer. All samples were transported to the laboratory on ice and stored at –20 °C until further analysis.

#### 4.2.3. Hydrogen and Oxygen Stable Isotope Analysis

Plant xylem water and soil water were extracted using an automated vacuum-condensation extraction system (LI-2100, Beijing, China), with each extraction lasting approximately 3 h and achieving an efficiency of >98%.

The hydrogen and oxygen isotope ratios (*δ*^2^H and *δ*^18^O) of the extracted water samples were determined using a Triple Isotope Water Analyzer (TIWA-45-EP, LGR, USA), which provided analytical precisions of ±0.5‰ for *δ*^2^H and ±0.1‰ for *δ*^18^O [8]. Each sample was measured six times, and the data were processed with LWIA Post Analysis software (v. 4.5.0.6). To minimize the carry-over effect of the injection system, the first two readings were discarded, and the average of the remaining four measurements was used for each sample. All isotope values are expressed in per mil (‰) relative to the Vienna Standard Mean Ocean Water (VSMOW). To minimize cross-contamination, no more than 18 samples were analyzed per batch.

It should be noted that low-temperature vacuum distillation of plant xylem water may co-distill volatile organic compounds (e.g., methanol and ethanol), which can interfere with spectroscopic detection and potentially bias the isotope results. To address this, the raw isotope data for plant xylem water were corrected using the integrated spectral-correction module of the LWIA Post Analysis software (v. 4.5.0.6).

Methanol spectral pollution correction curve:

Δ*δ*^18^O(y)~lnNB(x): y = 0.1455x^2^ + 0.0255x + 0.329 (1)

Δ*δ*^2^H(y)~lnNB(x): y = 0.2457x^2^ − 0.1651x + 0.6002 (2)

Ethanol spectral pollution correction curve:

Δ*δ*^18^O(y)~BB(x): y = −9.1411x + 9.1269 (3)

Δ*δ*^18^O and Δ*δ*^2^H are the deviations of the measured *δ*^18^O and *δ*^2^H values, respectively. NB and BB are the spectral measurements of the pollution degree of methanol and ethanol organic compounds determined by the spectral analysis software.

#### 4.2.4. Carbon Stable Isotope Analysis

The *δ*^13^C of each sample was determined by high-temperature combustion in an EA-ISOLINK elemental analyzer (Thermo Fisher Scientific, USA) to produce CO_2_, followed by isotope-ratio measurement using a Delta Q isotope ratio mass spectrometer (Thermo Fisher Scientific, USA). The *δ*^13^C was calibrated against the international reference standard Pee Dee Belemnite (PDB). Long-term WUE was subsequently calculated from the *δ*^13^C based on the formula established by Farquhar et al. (1989) for C_3_ plants [46]. Studies have also demonstrated that the *δ*^13^C values of plant leaves can also serve as a good indicator of WUE in C_4_ plants [47,48].

*C_a_* = 277.78 + 1.350exp(0.01572(*t* − 1740)) (4)

*δ*^13^*C_a_* = −6.429 − 0.006exp(0.0217(*t* − 1740)) (5)

(6) $$\Delta^{13}C=\frac{\delta^{13}C_{a}-\delta^{13}C_{p}}{1+\delta^{13}C/1000}$$

(7) $$\mathrm{WUE}=\frac{A}{g_{s}}=\frac{C_{a}-C_{i}}{1.6}=\frac{C_{a}(b-\Delta^{13}C)}{1.6(b-a)}$$

In the equation, t denotes the sampling year, *C*_a_ represents atmospheric CO_2_ concentration (μmol·mol^−1^), Δ^13^C indicates the difference in stable carbon isotope ratios (*δ*^13^C) of plant leaves, 1.6 is the diffusion ratio between water vapor and CO_2_ in air, a = 4.4 represents the diffusion fractionation coefficient of CO_2_ through stomata, and b = 27 denotes the fractionation coefficient during CO_2_ carboxylation by the Rubisco enzyme.

#### 4.2.5. Determination of Soil Water Content (SWC)

SWC was determined by the oven-drying method at 105 °C to constant weight, following the procedure described in Soil Agrochemical Analysis [49].

#### 4.2.6. Statistical Analysis

MixSIAR is a Bayesian mixture model implemented in R, designed to estimate the proportion of different potential water sources utilized by plants. Its key advantages include the simultaneous processing of multiple isotope datasets and the capacity to analyze diverse water sources, alongside operational efficiency and the quantification of estimation uncertainty, which collectively enhance accuracy and analytical rigor. It can be expressed by the following formula:

(8) $$\begin{array}{l} X_{\mathrm{ij}}=\sum_{k=1}^{k}P_{k}(S_{\mathrm{jk}}+C_{\mathrm{jk}})+\varepsilon_{\mathrm{ij}}\\ S_{\mathrm{jk}}\sim N(\mu_{\mathrm{JK}},\varpi_{\mathrm{jk}}^{2})\\ C_{\mathrm{jk}}\sim N(\lambda_{\mathrm{JK}},\tau_{\mathrm{jk}}^{2})\\ \varepsilon_{\mathrm{ij}}\sim N(0,\delta_{j}^{2}) \end{array}$$

In the MixSIAR model, *X*_ij_ denotes the isotope value j for a given mixture i (i.e., the isotopic value of plant xylem water), where i = 1, 2, 3, …, N and j = 1, 2, 3, …, J. *S*_jk_ denotes the source value k based on isotope value *j* (k = 1, 2, 3, …, K), following a normal distribution with mean *μ*_jk_ and standard deviation *ω*_jk_. *P*_k_ represents the proportion of source k estimated by the model. *C*_jk_ denotes the fractionation coefficient of isotope j in source k, following a normal distribution with mean *λ*_jk_ and standard deviation *τ*_jk_. *ε*_ij_ denotes the unquantified residual representing unquantified variations between individual mixtures, following a normal distribution with mean 0 and standard deviation *σ*_j_. During model operation, the hydrogen and oxygen isotope values of xylem water from different sand-fixing plants were input as mixed data into the MixSIAR model. The average hydrogen and oxygen isotopes of different soil layers (0–20 cm, 20–40 cm, 40–60 cm, 60–80 cm, 80–100 cm, 100–150 cm) were input as source data into the MixSIAR model. Since plants do not undergo isotope fractionation during water uptake, the mean and standard deviation of each potential source were set to 0 and input as differential data into the MixSIAR model. The Monte Carlo Markov chain run length was set to “long,” the error term selection was set to “poor,” and then the model was run.

The Water Niche Breadth index is used to quantify the diversity of resource utilization by species. Based on the concept of “resource units,” this index measures the species’ utilization distribution across different resource states to assess its niche breadth.

(9) $$B_{i}=\frac{1}{\sum_{i=1}^{n}{P_{\mathrm{ij}}}^{2}}$$

where *B*_i_ denotes the water niche breadth of species *i*; *n* denotes the total number of soil layers; and *P*_ij_ denotes the contribution rate of soil layer j to species *i*.

The Proportion Similarity Index (PS) is a metric characterizing ecological niche overlap between organisms. A higher PS value indicates greater niche overlap between two species and more intense competition for a given resource, whereas a lower PS value indicates minimal overlap and reduced competition. When species exhibit complete overlap in water sources, the PS value reaches its maximum (100%), with its theoretical range being [0, 1]. In this study, the PS index was employed to evaluate competition for water resources among different species.

(10) $$\mathrm{PS}=1-0.5\sum_{i=1}^{n}\left|P_{1i}-P_{2i}\right|$$

In this equation, PS denotes the Proportion Similarity Index, while P_1i_ and P_2i_ represent the utilization proportions of the i water source for any two species being compared.

Data were summarized using Excel 2021 and statistically analyzed with SPSS 27.0 software. Univariate analysis of variance (ANOVA) was used to determine variations in SWC among different planting years. Two-way ANOVA was used to examine the effects of planting year, species, and their interaction on soil water *δ*^18^O, plant xylem water *δ*^18^O, and plant WUE. Statistical significance was determined at *p* = 0.05. Plant water source contribution rates were calculated using the MixSIAR (v. 3.1.12) package in R (v. 4.5.0). Graphs were generated using R (v. 4.5.0) and Origin 2025b.

## 5. Conclusions

This study integrated stable isotope techniques with the MixSIAR model to investigate the water-use patterns and plasticity of three sand-fixing shelterbelt species. The results revealed significant interspecific differences in water sources among the three species, which also varied with stand age. With increasing planting year, *C. arborescens* exhibited the most pronounced shift in water uptake depth, indicating the greatest plasticity in water-use strategies. Long-term WUE differed significantly among species, and its relationship with water-niche breadth showed a negative trend, suggesting that broader water-niche breadth may be associated with lower WUE. The degree of water-source overlap among species declined in the later stages of the chronosequence. Collectively, these findings indicate that in hyperarid regions, desert plants can adjust their water-use strategies in response to stand age and water availability, while also showing shifts in water uptake depth consistent with water-source partitioning. This provides important insights for the sustainable construction of shelterbelt systems in arid environments. Accordingly, it is recommended that species selection and configuration may benefit from considering the water-use characteristics and temporal dynamics of each species, particularly combinations with lower water-source overlap, so as to enhance overall community WUE and stability.

## Data Availability

The original contributions presented in this study are included in the article. Further inquiries can be directed to the corresponding author.

## Abbreviations

The following abbreviations are used in this manuscript:

WUE	Water use efficiency
SWC	Soil water content
PS	Proportion Similarity Index
